# Levels of Exhaled Fraction of Nitric Oxide (FeNO) and Type 2 Biomarkers in Individuals Naturally Exposed to Helminth Parasites in a Tropical Region

**DOI:** 10.3390/ijms26178344

**Published:** 2025-08-28

**Authors:** Maria M. De Vivero, Randy Reina, Jonathan Ramírez, Josefina Zakzuk, Jose Miguel Escamilla Gil, Bayron Zelaya, Lucila Teresa Florez de Arco, Daniel P. Potaczek, Luis Caraballo, Nathalie Acevedo

**Affiliations:** 1 Institute for Immunological Research, University of Cartagena, Cartagena 130014, Colombia; mdeviverot@unicartagena.edu.co (M.M.D.V.); rreinar@unicartagena.edu.co (R.R.); jonkathan@gmail.com (J.R.); jzakzuks@unicartagena.edu.co (J.Z.); bayron.zelaya@unah.edu.hn (B.Z.); lcaraballog@unicartagena.edu.co (L.C.); 2 Clinica Respiratoria y de Alergias, Cartagena 130014, Colombia; jescamillagil@gmail.com (J.M.E.G.); lucilateresa@hotmail.com (L.T.F.d.A.); 3 Translational Inflammation Research Division & Core Facility for Single Cell Multiomics, Medical Faculty, Philipps-University of Marburg, 35043 Marburg, Germany; daniel.potaczek@staff.uni-marburg.de; 4 Center for Infections and Genomics of the Lung (CIGL), Justus Liebig University, 35390 Giessen, Germany

**Keywords:** exhaled fraction of nitric oxide, FeNO, helminth, asthma, infection, biomarkers, eosinophils, ascaris, rural, urban, IgE, CXCL1, CXCL9, SCF, type 2 inflammation

## Abstract

The exhaled fraction of nitric oxide (FeNO) is a biomarker of type 2 inflammation, reflecting the activity of inducible nitric oxide synthase (iNOS) in the bronchial epithelium in response to IL-4 and IL-13. Elevated FeNO levels support asthma diagnosis; however, it is unclear whether active helminth infections and rural environments influence this biomarker. The aim of this study was to compare FeNO levels among subjects naturally infected with helminth parasites and to evaluate their correlation with eosinophil counts and other inflammatory mediators. A total of 275 adult asthmatic patients and 161 healthy controls were involved; also, 223 asthmatic children and 114 healthy controls from the urban area of Cartagena were compared to 90 healthy children from a rural area. We found significant differences in FeNO levels between asthmatic patients and healthy controls in both adult and children’s cohorts (*p* < 0.0001). There was no difference in FeNO levels between Ascaris-positive and Ascaris-negative adults nor between subjects with active helminth infection and the non-infected. However, FeNO levels were significantly lower in rural healthy children (median 7.50 ppb, [IQR 4–14 ppb]) compared to urban healthy children (median 13.5 ppb, [IQR 10–18.5 ppb], *p* < 0.0001) and asthmatic children (median 20 ppb, [IQR 11–51 ppb], *p* < 0.0001). Rural healthy children had the highest total IgE levels (median 508 kU/L, [IQR 168–1020 kU/L]), high eosinophil counts (median 550 eos/μL, [IQR 360–800 eos/μL]) and plasma IL-5 levels (median 0.276 pg/mL, [IQR 0.19–0.53 pg/mL]). In conclusion, FeNO levels are not influenced by either natural exposure to helminth parasites or active infection, which supports its usefulness as a robust asthma biomarker in the tropics. Rural children have the lowest FeNO levels together with the highest total IgE levels, IL-5, and eosinophil counts, suggesting that lung-specific mechanisms are in place controlling iNOS expression during type 2 responses in healthy children.

## 1. Introduction

Helminth infections, mainly by *Ascaris lumbricoides*, are endemic in tropical developing countries and induce an intense type 2 immune response with high production of immunoglobulin (IgE) antibodies, which resembles in several molecular and physiological aspects the type 2 inflammation involved in the mechanisms of allergy and asthma [1,2]. Type 2 response is implicated in the defence against helminths [3], and alarmins, type 2 innate lymphoid cells (ILC2) [4], T helper 2 (Th2) cells, mast cells, and eosinophils help to expel worms and promote strong immunity to avoid reinfection [5,6]. Pulmonary type 2 response has also been shown to decrease trafficking of *Ascaris suum* larvae in lung tissue [7]. However, chronic and intense helminth infections induce host immunoregulation, suppressing the capacity to react to antigens/allergens and reducing the susceptibility to chronic inflammatory diseases including asthma [8,9,10,11,12,13]. This immunosuppression due to helminth infection has a gradient, and is not as strong in light infections, normally found in urban populations of underdeveloped areas [12,14]. Indeed, a number of studies have demonstrated that mild helminth infections can increase the predisposition to wheezing [15], bronchial hyperreactivity [16], asthma [17,18], and IgE sensitisation to invertebrate pan allergens [14,19,20].

To date, it is unclear if helminth infections could influence the levels and the cut-offs for the interpretation of type 2 biomarkers in tropical regions. We previously found that active *Ascaris lumbricoides* infection (ascariasis) is associated with decreased H3 and H4 histone acetylation at the promoter of the IL-13 encoding gene (*IL13*) poising its expression [21]. The presence of specific IgE antibodies to this helminth was also associated with H4 acetylation levels at the promoter of the *IL4* gene, encoding for IL-4 [21]. These cytokines are, however, difficult to measure in plasma, limiting their use as biomarkers. Instead, the exhaled fraction of nitric oxide (FeNO), IgE levels, and blood eosinophil counts are the most commonly used biomarkers of type 2 response and helpful to identify type 2 inflammation [22].

FeNO is a biomarker of type 2 inflammation that reflects the activity of the enzyme nitric oxide synthase (iNOS) in the bronchial epithelium in response to IL-4 and IL-13 [23]. FeNO levels above 25 parts per billion (ppb) in adults and 20 (ppb) in children are indicators of asthma [24] and have been included in recent guidelines for asthma diagnosis, at levels above 40 ppb in the GEMA guideline [25] and above 50 ppb in the GINA guideline (www.ginaasthma.org (accessed on 23 July 2025)). A previous study suggested that ascariasis was associated with elevated FeNO [26]. However, it is still unclear whether active helminth infection and rural environments influence FeNO levels in asthmatic children and adults. Helminth infections such as *A. lumbricoides*, which have a pulmonary phase in their life cycle [27], can cause a dry cough, wheezing, dyspnea, pulmonary infiltrates, and eosinophilia [28], and cause pneumonia and asthma-like symptoms in Loeffler syndrome [29]. Also, *A. lumbricoides* infection can modify plasma immune mediators and type 2 proteins [30] and cytokine levels [31]. For instance, periostin and chitinase-like protein YLK-40, the latter also known as chitinase 3 like 1 (CHI3L1), are recognised as asthma biomarkers in developed regions [32,33], but few studies have evaluated their levels in populations exposed to tropical parasites. As shown in a meta-analysis performed by Izuhara et al. [34], serum periostin levels correlate with blood eosinophil counts, FeNO, serum total IgE, and the response to anti IL-4/IL-13 therapies [33]. Moreover, an association between FeNO and periostin has been described [35,36]. YLK-40 is an acute phase protein associated with increased risk of asthma [32,37]. Mammalian chitinases and chitinase-like proteins are produced by neutrophils, monocytes, and macrophages in response to parasitic or fungal infections. Some studies have reported increased YKL-40 in children with *Schistosoma haematobium* infection [38] and changes in histone acetylation at the promoter of the YLK-40-encoding gene (*CHI3L1*) have been associated with specific IgE levels to Ascaris [21]. It is possible that helminth infections may affect the levels of these type 2 biomarkers or others plasma mediators.

The aims of this study were (1) to evaluate the relationship between FeNO and the presence of IgE antibodies against Ascaris, (2) to evaluate the effect of ascariasis on the levels of type 2 inflammation biomarkers in asthmatic patients, and (3) to evaluate the relationship of FeNO and eosinophil counts in asthmatic patients and healthy controls.

## 2. Results

### 2.1. FeNO Levels in Asthmatics with Positive Specific IgE to Ascaris

**Figure 1:**
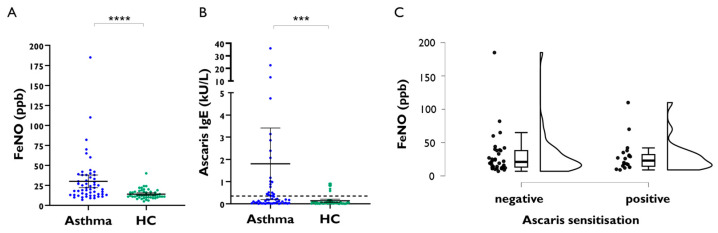
Comparative FeNO levels between adult asthmatic patients and healthy controls (HC) depending on the positive IgE sensitisation to the nematode Ascaris (p1 antigen, ImmunoCAP™). (**A**) FeNO levels between asthma patients and healthy controls. (**B**) Specific IgE levels to Ascaris between asthmatic patients and healthy controls. (**C**) FeNO levels according to the presence of positive IgE sensitisation to Ascaris in adult asthmatics. ppb: parts per billion. *p*-values are shown for significant differences. **** *p* < 0.0001, *** *p* = 0.003.

In an exploratory phase, FeNO levels were comparatively analysed in a total of 102 adult subjects, including 54 asthmatics and 48 healthy controls. FeNO levels were significantly higher in asthmatic patients compared to healthy controls (*p* < 0.0001), with a mean of 30 ppb [95%CI 22–38 ppb] in asthmatics and 14 ppb [95%CI, 12–16 ppb] in healthy subjects (Figure 1A). Specific IgE levels to Ascaris (p1 antigen, ImmunoCAP™) were also significantly higher in asthmatics (mean 1.8 kU/L [95%CI, 0.19–3.4 kU/L]) compared to controls (mean 0.13 kU/L [95%CI, 0.06–0.21 kU/L]) (Mann–Whitney *p* = 0.0005) (Figure 1B). There were 26 individuals with positive IgE to Ascaris (≥0.35 kU/L), and the frequency of positive individuals was higher in the asthma group (20.3%) compared to controls (*n* = 6, 12.5%), (*p* = 0.005). However, there was no difference in FeNO levels between asthmatics with positive IgE to Ascaris compared to those IgE-negative to Ascaris (*p* = 0.8; Figure 1C), even after adjusting for age and gender; also, there was no correlation between specific IgE levels and FeNO levels.

### 2.2. The Relationship of FeNO Levels and Specific IgE to Ascaris with Plasma Mediators in Asthmatics

In the subgroup of 54 adult asthmatics, we analysed the correlation between FeNO levels and plasma levels of immune mediators (including periostin, YKL-40, and 67 other inflammatory proteins). We found that FeNO levels were inversely correlated with levels of caspase 8, extracellular newly identified receptor for advanced glycation end-products binding protein (EN-RAGE), and sirtuin 2 (Supplementary file S1, Table S1). Also, there was a direct association between FeNO levels and periostin levels that was significant after adjusting by age and gender (Supplementary file S1, Table S2).

**Figure 2:**
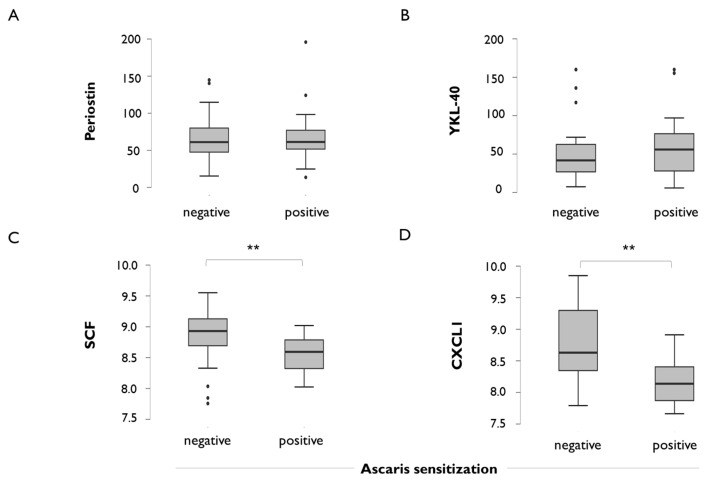
Plasma levels of inflammatory mediators in adult asthma patients according to the positive IgE sensitisation to the nematode Ascaris. (**A**) Periostin, (**B**) chitinase-3-like protein 1 (YKL-40), (**C**) stem cell factor (SCF), (**D**) CXC motif chemokine ligand 1 (CXCL1). Protein levels are expressed in Normalised Protein eXpression values (NPX); error bars indicate mean and 95% confidence interval. ** *p* = 0.01. *p*-values are shown for significant differences.

We then analysed the levels of immune mediators between Ascaris-positive and Ascaris-negative patients, and we found no differences in periostin (Figure 2A) or YKL-40 (Figure 2B) levels. Interestingly, two plasma proteins, stem cell factor (SCF) and CXC motif chemokine ligand 1 (CXCL1), were found significantly decreased in Ascaris-positive asthmatics (Figure 2C,D) and these differences remained significant after adjustment by age and gender. Overall, there was no relationship between plasma mediators associated with FeNO levels and those associated with specific IgE to Ascaris (Supplementary file S1, Table S3).

### 2.3. FeNO Levels in Adult Asthmatics with Positive Specific IgE to Ascaris lumbricoides and in the Context of Active Infection

**Figure 3:**
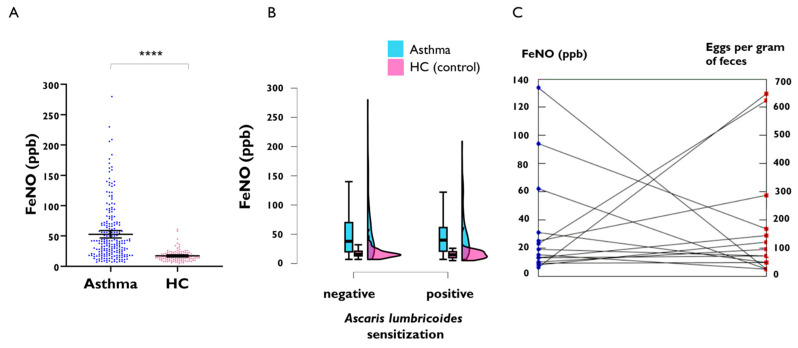
FeNO levels in a replication cohort of adult asthmatic patients and healthy controls (HC). (**A**) FeNO levels between asthma patients and healthy controls, **** *p* < 0.0001. (**B**) FeNO levels according to the presence of positive IgE sensitisation to *Ascaris lumbricoides* stratifying by disease group. (**C**) FeNO levels in 14 subjects with active *Ascaris lumbricoides* infection. The line connects paired values for FeNO and eggs per gram (epg) of faeces in the same subject.

To further compare FeNO levels according to the presence of specific IgE to *Ascaris lumbricoides*, an independent cohort of 221 adult asthmatics and 113 healthy controls were analysed. FeNO levels were higher in asthmatic patients (mean, 52.8 ppb [95%CI, 46.7–58.9 ppb]) compared to healthy controls (mean, 17.3 ppb [95%CI 15.6–19.0 ppb]; Figure 3A), and there was no difference in FeNO levels between individuals with positive specific IgE to *Ascaris lumbricoides* in either the group of asthmatics or in healthy controls (Figure 3B). Since specific IgE reflects past exposure but not active infection, we then analysed the relationship between the presence of active *Ascaris lumbricoides* infection, as detected by stool analysis and FeNO levels. We only found 14 cases of active *Ascaris lumbricoides* infection in adults, specifically in seven asthmatics and seven controls, and we found that FeNO levels were not altered by active infection of light intensity (Figure 3C).

### 2.4. FeNO Levels in Asthmatics and Healthy Children with Active Ascaris lumbricoides Infection

**Figure 4:**
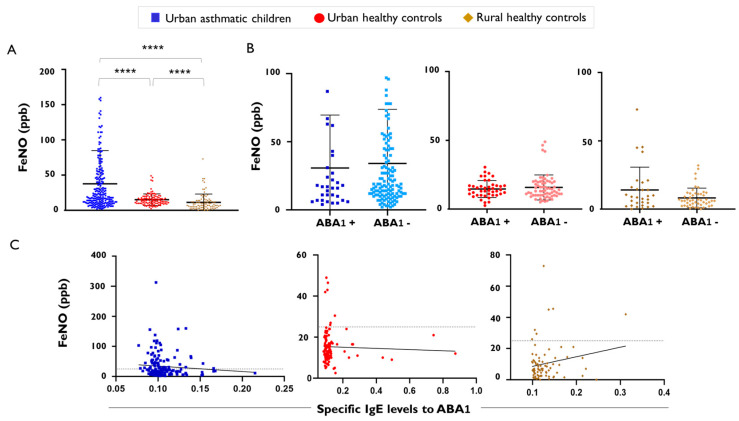
FeNO levels in asthmatic children and healthy controls. (**A**) FeNO levels between asthmatic children and healthy controls from urban and rural environments, **** *p* < 0.0001. (**B**) FeNO levels according to the positive IgE sensitisation to the Ascaris specific antigen ABA-1. Error bars indicate mean and standard deviation (**C**) Correlation between FeNO levels and IgE levels to the Ascaris specific antigen ABA-1 (in optical density); The black straight line indicates the line of best fit for the linear regression of FeNO and specific IgE levels to ABA-1. The dotted gray line indicates the cutoff of normal FeNO values.

We then compared FeNO levels in a paediatric cohort of asthmatic children and controls, and we observed significant differences in FeNO levels between asthmatic children (*n* = 223; median 20 ppb [IQR 11–51 ppb]) and healthy urban controls (*n* = 103; median 13.5 ppb [IQR 10–18.5 ppb]; Figure 4A). We also included a subset of rural healthy children to explore FeNO levels in this environment, in which helminth infections are more prevalent than in urban children. We found that FeNO levels were significantly lower in healthy rural children (*n* = 90; median 7.50 ppb [95%CI, IQR 4–14 ppb]) compared to urban healthy children or asthmatics (Figure 4A). In the asthmatic group, there were six children with active *A. lumbricoides* infection as diagnosed by stool test, and one of them was coinfected with *Trichuris trichiura* (138 epg of faeces). In the group of urban healthy children, we found nine infected with *A. lumbricoides*. Their FeNO values are presented in Table 1.

In the rural children, we found 17 children infected by *Trichuris trichiura*, 2 coinfected with *Ancylostoma duodenale*, 2 with *Hymenolepis nana*, and 2 with *Ascaris lumbricoides* (Table 2). The low number of children diagnosed with an active infection precluded statistical comparison of FeNO levels between infected and non-infected subjects. However, we compared FeNO levels according to the presence of positive IgE to the nematode-specific antigen ABA-1. We found no differences in FeNO levels between ABA-1-positive and ABA-1-negative children (Figure 4B). Moreover, we found no significant correlation between FeNO and IgE levels to ABA-1 in any of the groups analysed (Figure 4C).

### 2.5. Other Type 2 Biomarkers and Chemokines in Healthy Rural Children with Low FeNO Levels

**Figure 5:**
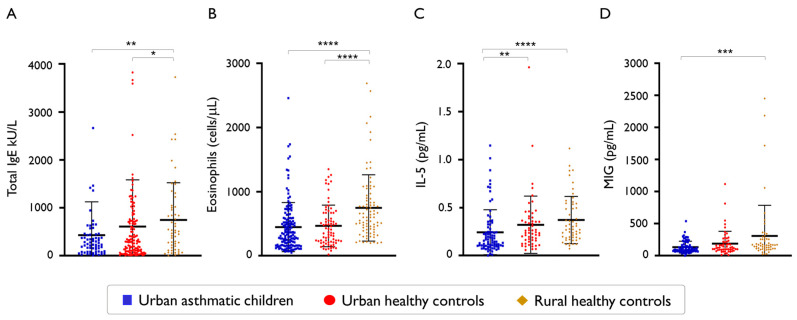
Type 2 inflammation biomarkers between asthmatic children and healthy controls from urban and rural environments. (**A**) Total IgE levels * *p* = 0.0361, ** *p* = 0.0032, (**B**) peripheral blood eosinophil counts **** *p* < 0.0001, (**C**) plasma IL-5 levels ** *p* = 0.0060, **** *p* < 0.0001, (**D**) Monokine Induced by Interferon-gamma (MIG) levels *** *p* = 0.0001. Error bars indicate mean and standard deviation.

We then compared total IgE levels and the numbers of peripheral blood eosinophil counts between groups. There was no significant difference in total IgE levels between asthmatic children (median, 245 kU/L IQR, 86–443 kU/L]) and urban healthy children (median, 292 kU/L [ IQR, 94–729 kU/L]; *p* = 0.35), but we found that total IgE was significantly higher in rural healthy children (median, 508 kU/L [IQR 168–1020 kU/L]) compared to asthmatics (*p* = 0.003) or urban healthy children (*p* = 0.03; Figure 5A). Regarding eosinophil cells counts, there was no difference between asthmatic children (median, 340 cells/μL [IQR 160–580 cells/μL]) and urban healthy children (median, 370 cells/μL [IQR 210–670 cells/μl]; *p* = 0.34), but those were significantly higher in rural healthy children (median, 600 cells/μL [IQR 410–897 cells/μL] compared to any of the other two groups (*p* < 0.0001), as shown in Figure 5B. Moreover, we found that plasma levels of IL-5 were significantly higher in urban healthy children (median, 0.24 pg/mL [IQR, 0.15–0.38 pg/mL]) compared to asthmatic children (median, 0.16 pg/mL [IQR 0.10–0.28 pg/mL]; *p* = 0.006), and even the highest in rural healthy children (median, 0.27 pg/mL [IQR 0.19–0.53 pg/mL]) compared to asthmatic children (*p* < 0.0001; Figure 5C), but there was no difference in IL-5 levels between healthy children from urban and rural environments as shown in Figure 5C. When we analysed chemokine levels, we found increased levels of CXCL9/MIG, a bioactive form of interferon gamma, in rural healthy children (median, 165 pg/mL [IQR, 111–262 pg/mL]) compared to asthmatic children (median, 100 pg/mL [IQR 69–166 pg/mL]; *p* = 0.0001; Figure 5D). CXCL9/MIG levels were also higher in healthy urban children than in asthmatic children, but this difference was not statistically significant (*p* = 0.062).

### 2.6. The Relationship of FeNO Levels with Blood Eosinophil Counts

**Figure 6:**
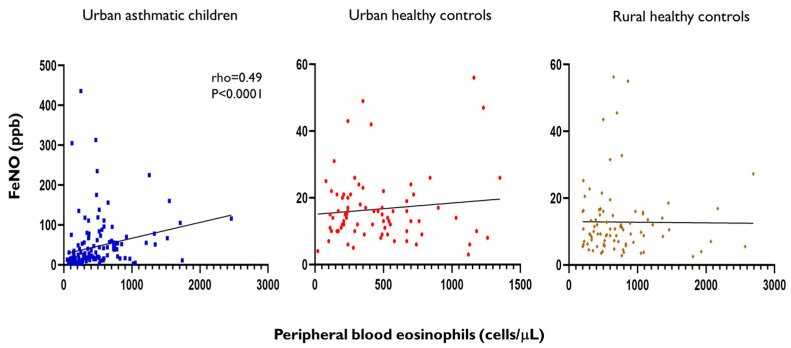
Spearman correlation between FeNO levels and blood eosinophil counts in asthmatic children and healthy controls from urban and rural environments. Each dot represents a child, coefficients and *p*-values are only shown for significant correlations. The black straight line indicates the line of best fit for the linear regression of FeNO levels and blood eosinophil counts.

We found significant positive correlation between FeNO levels and peripheral blood eosinophil counts in asthmatic children (Spearman rho = 0.49, *p* < 0.0001). However, FeNO levels were not correlated with blood eosinophil counts in healthy children from urban or rural environments (Spearman rho = −0.02, *p* = 0.80 in urban children and Spearman rho = −0–04, *p* = 0.69 in rural children) (Figure 6).

**Figure 7:**
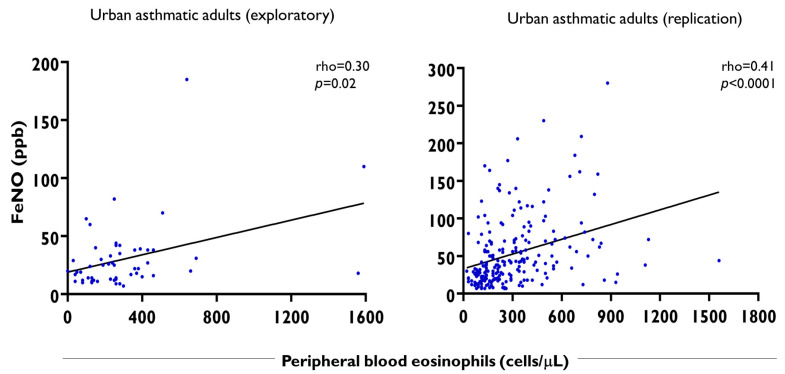
Spearman correlation between FeNO levels and peripheral blood eosinophil counts in adult asthmatics from two cohorts. Each dot represents an asthmatic patient. Eos/μL: peripheral blood eosinophil counts per microliter of blood. The black straight line indicates the line of best fit for the linear regression of FeNO levels and blood eosinophil counts.

In adult asthmatic patients, we also confirmed significant correlation between FeNO levels and blood eosinophil counts in both cohorts (Figure 7), and there was no correlation between FeNO levels and blood eosinophil counts in adult healthy controls.

**Figure 8:**
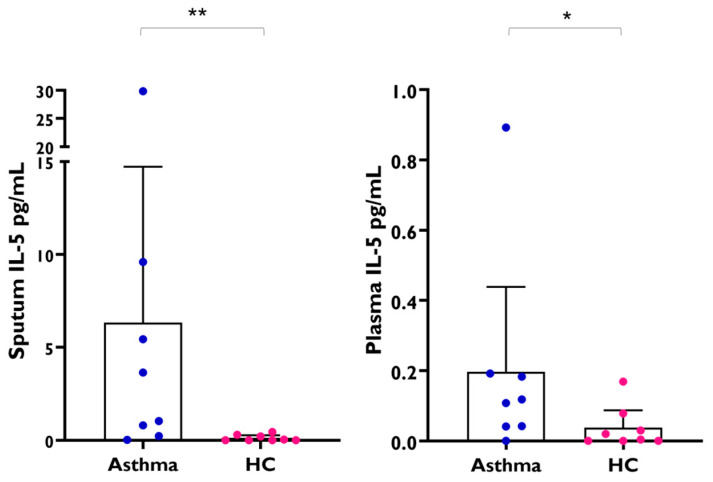
IL-5 levels in induced sputum samples and paired plasma samples from adult asthmatic patients (*n* = 8) and healthy controls (HC, *n* = 8). Each dot represents an individual, error bars indicate mean and 95% confidence interval. (Mann–Whitney test sputum IL-5, asthma vs. HC, ** *p* = 0.0042) (Mann–Whitney test plasma IL-5, asthma vs. HC, * *p* = 0.039).

To investigate further the correlation between FeNO and blood eosinophils only in asthmatic subjects and not in healthy subjects, we analysed IL-5 levels in induced sputum samples of adult asthmatics and healthy controls. We found that asthmatics have significant increased levels of IL-5 in sputum (mean 2.3 [95%CI 0.37–8.6]) compared to healthy subjects (mean 0.02, [95%CI 0.0–0.28], *p* = 0.004). IL-5 levels in plasma were also higher in adult asthmatic patients (mean 0.11 [95%CI 0.04–0.19]) compared to healthy controls (mean 0.012, [95%CI 0.0- 0.06], *p* = 0.039) (Figure 8); remarkably, IL-5 levels in sputum were higher than those detected in plasma.

## 3. Discussion

In this study, we report the levels of FeNO in adult asthmatic patients from an urban tropical area, and in a paediatric cohort of asthmatics and healthy children living in an urban or rural environment. We show that FeNO levels are significantly increased in asthmatic patients living in tropical areas and can be used as a robust asthma biomarker in our region, and that FeNO levels are not affected by past or active helminth infection by *Ascaris lumbricoides*. We also confirmed positive correlations of FeNO with blood eosinophil counts and FeNO with periostin levels in asthmatic patients [35,36].

Based on previous studies suggesting that ascariasis can influence asthma [26,39], we analysed the relationship between *A. lumbricoides* exposure, FeNO levels, and plasma immune mediators, finding that FeNO did not differ between Ascaris-positive and Ascaris-negative asthmatics, suggesting that it is not influenced by Ascaris sensitisation in adult patients. The levels of other type 2 biomarkers, such as periostin and YKL-40, also did not differ according to the presence of Ascaris sensitisation. However, lower plasma levels of SCF and CXCL1 were observed in Ascaris-positive asthmatics, suggesting that previous infection with this nematode can influence plasma levels of some immune mediators. These differences were still significant after adjusting for covariates.

SCF is a cytokine produced by several cell types and triggers its biological effects by binding to its receptor c-Kit, a member of the tyrosine kinase receptor family [40]. It plays a crucial role in allergic inflammation, particularly in the context of airway hyperreactivity and eosinophil recruitment [41,42]. It acts as a cytokine that promotes mast cell survival, activation, and differentiation, which in turn can lead to increased inflammation and allergic symptoms [43]. SCF also directly interacts with eosinophils, causing their activation and degranulation, leading to the release of inflammatory mediators and chemokines [44]. SCF is also implicated in the protective immune response against intestinal helminth infections [45]. SCF contributes to mucosal mast cell hyperplasia during nematode infection [40,46]. It has also been shown that administration of an anti-SCF polyclonal antibody completely eliminated the mucosal mast cell population in a rat model [46], impairing worm expulsion in animals infected with *T. spiralis* [47,48]. Other reports suggest that SCF can reduce type 2 inflammation [49]. Further studies are needed to elucidate the mechanisms of how Ascaris exposure reduces SCF levels in human plasma and if this is a consequence of host immunomodulation by this helminth.

We also observed reduced levels of CXCL1 in Ascaris-sensitised asthmatics. Chemokines are involved in type 2 inflammation in allergic diseases [50]. Indeed, during allergy and anti-helminth immunity, epithelial cells of damaged barriers release alarmins including IL-25, IL-33, and thymic stromal lymphopoietin (TSLP), but also chemokines such as CXCL1 and CCL11, to promote cell recruitment and inflammation [51]. CXCL1 is involved in airway inflammation, and its presence in the airways of asthmatic patients and animal models of allergy has been linked to increased levels of IL-17 and neutrophil activation [52,53]. In the context of infection, CXCL1 acts as a chemotactic signal, attracting neutrophils to the area where parasites are present [54]. The decrease in CXCL1 in asthma leads to the increased recruitment of mast cells to the airways [55]. In this study, we found decreased levels of SCF and CXCL1 in asthma patients with positive IgE to Ascaris. More studies are needed to understand how the reduction in SCF and CXCL1 observed in our patients occurred by natural exposure to Ascaris and its implications in their immune response [56].

We then confirmed that FeNO levels are higher in asthmatics than in healthy controls in an independent adult sample set. In addition, we replicated our observation made in the exploratory cohort that positive IgE to *Ascaris lumbricoides* does not influence FeNO levels. Since positive IgE reflects parasite exposure but not necessarily active infection, we also analysed FeNO levels in subjects with worm eggs in faeces. The presence of active infection by the nematode *Ascaris lumbricoides* did not increase FeNO levels (Figure 3C and Table 1 and Table 2).

We then analysed FeNO levels and type 2 biomarkers in a paediatric cohort, in which stool samples were collected and analyses for parasitic infection were performed. We confirmed that FeNO levels were higher in asthmatic children and that FeNO levels were not influenced by natural infection by helminth parasites in children, which supports its usefulness as a robust asthma biomarker in the tropics. There were also other types of parasites detected, such as *Trichuris trichiura*, which also did not affect FeNO levels. Collectively, it might suggest that even though these nematodes induce a systemic type 2 immune response, those are not sufficient to generate a local response in the lung capable of altering FeNO levels. We focused mainly on *Ascaris lumbricoides* because it undergoes a migratory phase through the lungs in its life cycle [27], during which it induces strong tissue eosinophilic inflammation. This infection may cause cough and wheezing and is known as Loeffler syndrome [29]. In the rural cohort, the infections with *Trichuris trichiura* were more common than those with *Ascaris lumbricoides*, and we could observe that FeNO levels were not affected. Unlike *Ascaris lumbricoides*, *Trichuris trichiura* does not undergo a pulmonary phase [57]. Moreover, it should be considered that the numbers of eggs per gram of faeces in our samples were low, indicating infections of light intensity. We cannot rule out that, in the case of heavy intensity infection (>50,000 eggs), the response may differ, as was found in a work performed in a small village of Colombia [39].

Depending on intensity of the infection and type of helminth [58], some helminthiasis can downregulate different immune pathways [59], being helminths with life stages in different tissues and/or solid organs (i.e., *Schistosoma* ssp., *Ancylostoma duodenale*, *Trichinella* ssp.) very suppressive [60,61]. *Ascaris lumbricoides* also have several immunomodulatory molecules in their body fluids [62,63,64] and can modulate the immune responses [8,30,39,65]. Also, early heavy infections with *T. trichiura* may protect against the development of allergen skin test reactivity in later childhood [66].

We also confirmed no difference in FeNO levels between children with positive IgE levels to ABA-1 and the negative controls in any of the analysed groups. Interestingly, rural children had the lowest FeNO levels while, at the same time, they had the highest total IgE levels, blood eosinophil counts, and plasma IL-5 levels, suggesting that lung-specific mechanisms are in place that control lung iNOS expression during systemic type 2 response in healthy children. The elevated production of MIG/CXCL9, a bioactive form of interferon gamma, in rural children may play a role in their immune homeostasis. The increased levels of IL-5 in rural children, in whom helminth infections are more frequent, reflects the importance of this cytokine in helminth response. However, in the context of a respiratory disease such as asthma, bronchial IL-5 levels increase and blood levels do not reflect that found in the airways. Although several factors may influence immune responses in rural children, the apparent dissociation of high systemic type 2 markers (IgE, eosinophils, IL-5) but the lowest FeNO can be explained; first by helminth infections that favour local lung regulatory mechanisms [67] and downregulation of the IL-4/IL-13 pathway [68,69] and thereby iNOS, together with protozoa coinfections that boost type 1 and type 17 responses. Moreover, helminth infections are known to modify arginase activity in peripheral blood mononuclear cells and plasma [70] and may affect availability of L-arginine needed by iNOS to produce nitric oxide.

The comparison of IL-5 levels in plasma and induced sputum samples revealed that the degree and intensity of IL-5 response differs between plasma and the airways, being below 1 pg/mL in plasma, in the setting of natural exposure to helminths but much higher in the airway as measured in the induced sputum of adult asthmatic patients. Although type 2 responses, as reflected by higher IL-5 in plasma, were even stronger in healthy rural children compared to urban healthy or asthmatic children, the measured plasma IL-5 levels were much lower than those observed in the sputum of adult asthmatics. Positive correlations between blood eosinophils counts and FeNO levels, observed in all cohorts of asthmatics but not in healthy controls, illustrates the dysregulation of IL-5 in asthma [71] and the relevance of this pathway in asthma therapy [72]. These findings also indicate that, in addition to analysing peripheral blood eosinophils counts, future studies are needed to elucidate their cell surface markers and subtypes, identifying those that are relevant in helminth infections [73] or in disease states like asthma. The magnitude of correlation between FeNO levels and blood eosinophil counts also reflects that these two markers although directly related, are providing information on two different type 2 inflammation pathways.

It also important to consider the species-specific effects that each helminth may exert on asthma. It has been reported that *Trichuris trichiura* infections during the first 5 years of life were associated with reduced wheeze, while *Ascaris lumbricoides* infestations at 5 years of age were related to increased wheeze and asthma [26]. Recent meta-analyses suggest that Ascaris can increase the risk of bronchial hyperreactivity [16], whereas other studies suggest that helminth effects on asthma and wheeze can be protective [74]. Thus, the data are conflicting. In this study, we confirmed that natural exposure to *Ascaris lumbricoides*, even though it undergoes a pulmonary migration phase, does not affect FeNO levels in humans, and that there is a dissociation between type 2 responses occurring during natural helminth exposure not accompanied by asthma and type 2 inflammation during asthma.

## 4. Materials and Methods

### 4.1. Adult Cohorts

We included two adult cohorts of asthmatic patients and healthy controls. The first and exploratory dataset comprised 102 individuals from Cartagena (Colombia), including 54 adult asthma patients and 48 healthy controls. This subset of patients was recruited in a specialised pulmonary clinic and diagnosed by a pulmonologist [75] following the GEMA criteria and pulmonary function tests [25]. The second and independent dataset consisting of 221 adult asthmatics and 113 healthy controls from the “Study on the Pathogenesis of Asthma in the Tropics (EPAT)”, with data on stool analysis and IgE levels to *A. lumbricoides*, was used as a replication cohort. All participants signed a written informed consent to participate in the study. This study was approved by the Ethics Committee of the University of Cartagena (No. 416-9722017 and No. 128-14112019).

### 4.2. Children Cohort

#### 4.2.1. Urban Children

A total of 338 urban children were included, including 223 asthmatics and 114 healthy controls. Asthmatic children were recruited in a specialised local clinic in which the diagnosis of asthma was performed by an allergologist or pulmonologist. Children and their parents were reinterviewed and examined by a physician of the research staff. Demographic and medical information was documented by questionaries. The healthy children were recruited in local schools and neighbourhoods. Mothers were questioned about health conditions, familiar history of disease, and exposures. Parents or legal guardians signed a written informed consent to participate.

#### 4.2.2. Rural Children

Samples from rural children were collected by random sampling in a rural area of Santa Catalina, Colorado, and Loma Arena, three small villages of the department of Bolívar, contacting approximately 100 children. Of these, 90 children were eligible with stool sample to detect active parasite infections and assess FeNO levels. A subset of 55 children with available blood samples were included for protein plasma measurements. The children were 6–14 years old, so they were capable of performing the FeNO measurement. This group was classified into rural children with a parasitic infection diagnosed by stool examination (infected group) and rural children with a negative stool examination and no clinical signs of intestinal parasitosis (non-infected group). Parents or legal guardians signed a written informed consent to participate.

### 4.3. Fractional Exhaled Nitric Oxide (FeNO)

FeNO measurement was performed by a researcher trained in this procedure, using the portable NObreath^®^ device v2. (Bedfont Scientific Ltd, Kent, UK). Three measurements were taken per patient at a flow rate of 50 mL/s, monitored by a visible airflow metre and following the recommendations of the American Thoracic Society [24]. Results were expressed in parts per billion (ppb). Prior to measuring FeNO, each individual was questioned about recent cigarette smoking, complete fasting, and whether they had had respiratory infections in the past 2 months. FeNO measurements for this study were recorded for those not having smoked cigarettes in the previous 48 h, having fasted for at least 12 h, not having consumed alcohol, or reported no symptoms of respiratory infections in the past 2 months. FeNO levels are considered normal below <25 ppb in adults and <20 ppb in children [24]. According to the GINA guideline, FeNO values above 50 ppb in adults and 35 ppb in children support the diagnosis of type 2 asthma (Global Initiative for Asthma. Global Strategy for Asthma Management and Prevention 2025. Updated May 2025, available from: www.ginaasthma.org (accessed on 23 July 2025)).

### 4.4. Blood Sample Collection

Blood samples were collected in EDTA tubes by antecubital venipuncture according to standardised protocols. A tube was used to measure white blood cell counts (WBC) including eosinophils in an automated haematology analyser (Advia 2120i, Siemens Healthcare Diagnostics Inc., Tarrytown, NY, USA), and another tube was centrifuged at 1000 rpm and 4 °C to obtain plasma. This was carefully separated with sterile micropipette tips, aliquoted into sterile tubes, and stored at −70 °C until use.

### 4.5. Stool Samples

Stool samples were collected by children’s mothers in a sterile plastic provided by the researchers and analysed using 0.85% saline solution and Lugol’s stain. If parasites were found, egg quantification was performed using the Kato–Katz method using a commercial kit (Copro Kit, C&M Medical, Campinas, Brazil). The helminth load was expressed as eggs per gram (epg) of faeces. The presence of protozoa, helminth eggs, or adult parasites was diagnostic of active infection (positive/negative). *A. lumbricoides* infection was classified as light intensity (1–4999 eggs), moderate intensity (5000–49,999), and heavy intensity (>50,000 eggs), following criteria of the World Health organisation (WHO) [76].

### 4.6. Induced Sputum Samples

Sputum was collected from 16 subjects (8 asthmatics and 8 healthy controls) using nebulised 4% saline solution and dissolved using 10% DTT (SPUTOLYSIN^®®^, EMD Millipore Corp, Burlington, MA, USA). After homogenisation for 15 min at 37 °C samples were centrifuged at 400× *g* for 10 min at 4 °C, and supernatant was collected in sterile tubes and stored at −70 °C until cytokine measurement, as described previously [77,78].

### 4.7. IgE Measurements

Total IgE levels were measured by ImmunoCAP™ (Thermo Fisher, Uppsala, Sweden) in all the participants; a calibration curve was used with a range of 2 to 5000 kU/L (2, 10, 50, 200, 1000, 5000 kU/L). Specific IgE levels to *Ascaris* spp. (p1) were measured in the adult cohort by ImmunoCAP™, a calibration curve of 0.35 to 100 kUA/L was used (0.35, 0.7, 3.5, 17.5, 50, 100 kUA/L). A cut-off of 0.35 kUA/L was used to consider a positive IgE sensitisation to Ascaris. Specific IgE levels to *A. lumbricoides* and the nematode specific marker ABA-1 were quantified using enzyme-linked immunosorbent assay (ELISA) in the EPAT cohort and the children cohort. Antigens were diluted in PBS and 64 mM NaHCO_3_-Na_2_CO_3_ buffer (pH 9.6). Each well was coated with 1μg of antigen, incubated overnight at 4 °C, washed five times with 0.1% PBS-Tween 20, and then blocked for 3 h using 1% Bovine Serum Albumin (BSA) in humid chamber at room temperature. After five washes, serum samples (diluted 1:5) were added to each well and incubated overnight at 4 °C in humid chamber. After five washes, plates were incubated for 2 h with antihuman IgE–alkaline phosphatase conjugate (Sigma-Aldrich Co., St. Louis, MO, USA) at room temperature. The assay was developed with *para*-nitrophenyl phosphate (1 mg/mL, Sigma-Aldrich, Co., St. Louis, MO, USA) diluted in 10% diethanolamine (pH 9.8) and the plate incubated in the dark at room temperature for 30 min. The reaction was stopped with 100 μl of 3 N NaOH per well and absorbance measured at 405 nm using a spectrophotometer (MultiScan Go, Thermo Fisher Scientific, Vantaa, Finland). Determinations were made in duplicate, and IgE levels above 0.113 optical density (mean OD of 6 negative, non-allergic, non-parasite-exposed controls + 3 SD) were considered positive for the presence of specific IgE to Ascaris or ABA-1.

### 4.8. Periostin and YKL-40 Measurements

Plasma periostin/OSF-2 levels were measured using a sandwich enzyme-linked immunosorbent assay (ELISA) with a capture antibody in the solid phase (Cat. 844441, R&D Systems, Inc, Minneapolis, MN, USA) and detection antibodies (Cat. 844442, R&D Systems, Inc, Minneapolis, MN, USA), using a DuoSet^®®^ 2 kit (R&D Systems, Inc, Minneapolis, MN, USA, Cat. DY008) as previously described [75]. YKL-40 measurement was performed with a commercially ELISA Duoset kit (Cat. DY2599 R&D Systems, Minneapolis, MN, USA) following the manufacturer’s instructions and the levels were expressed in pg/mL.

### 4.9. Proximity Extension Assay for Plasma Protein Levels

For plasma profiling in the adult cohort, samples were randomly distributed in 96-well plates, and protein levels were measured by Proximity Extension Assay (PEA) using the Target 96 Inflammation Panel (Olink Proteomics, Analysis Service Facility, Boston, USA), which includes a broad selection of proteins established as inflammatory signatures in diverse inflammatory diseases. A total of 67 out of 92 plasma molecules were detected in heparinized plasma (73%). Normalised Protein Levels are expressed as NPX units (log2 scale). The intra- and inter-assay average coefficients of variation (%CV) were 6% and 11%, respectively.

### 4.10. Cytokine Bead Array

In the children cohort, plasma IL-5 and chemokine levels (IL-8, MCP-1, IP-10, MIG, RANTES) were measured by flow cytometry using the Cytokine Bead Array (CBA). IL-5 measurements were performed with the BD^TM^ CBA Human IL-5 Flex Set (BD Biosciences, San Diego, CA, USA). In brief, mixed capture beads were incubated with human plasma and standards for 1 h at room temperature, then the human IL-5 PE detection reagent was added and incubated for 2 h at room temperature in the dark, beads were washed and acquired. For chemokines, the BD^TM^ CBA Human Chemokine Kit (BD Biosciences, San Diego, CA, USA) was used. In brief, five bead populations were mixed together to form an array that is resolved in a red channel (FL3-FL4) with different fluorescence intensities ranging from the dimmest to the brightest. Recombinant standards or human plasma samples were incubated with capture beads and the PE-conjugated detection antibodies for 3 h at room temperature in the dark. Wash buffer was added and samples centrifuged at 200× *g* for 5 min; afterwards, supernatant was carefully removed and the beads resuspended in wash buffer. Samples were acquired in a BD FACSAria^TM^ III cell sorter (BD Biosciences, San Jose, CA, USA), with reporter channel in PE and bead channels in APC. The intensity of PE fluorescence of each sandwich complex was compared to a standard curve from 0 to 2500 pg/mL, with the four-parameter curve fit option to extrapolate values with the concentration of each chemokine. Theoretical limits of detection were 0.2 pg/mL for IL-8, 1 pg/mL for RANTES, 2.5 pg/mL for MIG/CXCL9, 2.7 pg/mL for MCP-1 and 2.8 pg/mL for IP-10, and 1.1 pg/mL for IL-5. The FCAP Array^TM^ software (v3.0.1) was used to generate results in concentrations derived from the standard curve.

### 4.11. Statistical Analysis

Qualitative variables were compared using chi2 test. Normality of the distribution of the qualitative variables was tested the Shapiro–Wilk method. Quantitative variables are given as means with 95% confidence interval (95%CI) or medians with interquartile ranges, depending on the distribution observed. Comparisons of qualitative variables between two groups were performed using the Mann–Whitney U-test. Correlations were analysed using the Spearman coefficient. Linear regression was used to model the relationship between FeNO levels, mediator levels, and specific IgE levels, adjusting by age and gender. A *p* value ≤ 0.05 was considered statistically significant. Statistical analysis was performed using GraphPad Prism (v8.0.1) and JASP (v0.19.3).

## 5. Conclusions

In conclusion, FeNO levels were significantly higher in asthmatic patients compared to healthy controls, and there were no differences in FeNO levels between Ascaris-positive and Ascaris-negative controls. FeNO levels were not increased in adults or children with active infection by *Ascaris lumbricoides*. In this study, we observed for the first time lower levels of SCF and CXCL1 in asthmatic patients with positive IgE levels to Ascaris. Under type 2 response, plasma IL-5 levels, and eosinophils were significantly higher in rural children than in asthmatic children, showing a coexistence of increased type 2 biomarkers in peripheral blood circulation with the lowest FeNO levels. Rural controls also showed the highest total IgE levels. The increase in circulating type 2 biomarkers in rural children coincided with increased CXCL9 levels. Considering high FeNO levels and the fact that IL-5 is detectable at much high concentration in the induced sputum of asthmatics, one might hypothesize that divergent control mechanisms of the type 2 responses in health and disease exists, with very strong type 2 responses in the lung of allergic asthmatics, and only a subtle fine-tuning of this local, pulmonary production of type 2 mediators by helminth infections otherwise associated with systemically enhanced type 2 responses. FeNO levels and peripheral blood eosinophils are positively correlated biomarkers, and both important as indicators of type 2 inflammation, however, FeNO is more robust to confirm lung type 2 inflammation in asthma, because blood eosinophil counts can be found increased in healthy children of the tropics (and especially those in rural environments).

## Figures and Tables

**Table 1:** FeNO levels in urban children with active Ascaris infection.

Code	Group	Age	Sex	FeNO (ppb)	Ascaris Epg of Faeces
Cs013	HC	7	M	10	72
Cs024	HC	5	F	12	322
Cs029	HC	10	F	13	2783
Cs031	HC	10	M	14	3657
Cs062	HC	11	F	11	4
Cs086	HC	6	F	15	192
Cs094	HC	8	F	20	120
Cs108	HC	8	M	22	1416
Cs142	HC	14	F	10	24
AN022	Asthma	12	F	18	96
AN047	Asthma	7	M	16	2277
AN057	Asthma	12	M	7	7636
AN223	Asthma	8	M	29	288
AN279	Asthma	9	M	5	96
AN338	Asthma	10	M	18	24

**Table 2:** FeNO levels in rural children with active helminth infection.

Code	Age	Sex	FeNO (ppb)	Trichuris Trichiura	Ascaris Lumbricoides	Ancylostoma Duodenale	Hymenolepis Nana
LA012	7	M	1	1872	72	-	-
LA019	7	M	16	120	-	-	-
LA021	8	M	45	72	-	-	-
LA028	8	F	4.5	600	-	-	-
LA034	8	M	0	48	-	168	-
LA041	9	F	2.5	2592	-	-	-
LA053	9	M	21	336	-	-	-
LA056	6	F	2	6480	-	-	-
LA059	11	M	3	480	-	-	-
LA060	7	M	2	17,520	-	-	-
LA061	7	M	5	360	-	-	-
ST050	6	M	7.5	216	-	-	-
ST085	13	F	6.5	96	-	-	-
ST093	9	F	4	120	-	-	72
ST094	7	M	12	48	-	312	72
ST095	8	F	19.5	288	1824	-	-
ST097	10	F	22.3	9432	-	-	-

For each helminth the number indicates eggs per gram of faeces.

## Data Availability

The raw data supporting the conclusions of this article will be made available by the authors on request.

## Supplementary Materials

The following supporting information can be downloaded at: https://www.mdpi.com/article/10.3390/ijms26178344/s1.

## Abbreviations

The following abbreviations are used in this manuscript:

CI	Confidence interval
CXCL1	C-X-C motif chemokine ligand 1
CXCL9	C-X-C motif chemokine ligand 9
epg	Eggs per gram
FeNO	Exhaled fraction of nitric oxide
GINA	Global Initiative for Asthma
HC	Healthy control
IL-5	Interleukin 5
iNOS	Inducible nitric oxide synthase
IQR	Interquartile range
ppb	Parts per billion
SCF	Stem cell factor
